# Binding and dynamics of melatonin at the interface of phosphatidylcholine-cholesterol membranes

**DOI:** 10.1371/journal.pone.0224624

**Published:** 2019-11-07

**Authors:** Huixia Lu, Jordi Martí

**Affiliations:** Department of Physics, Technical University of Catalonia-Barcelona Tech. Barcelona, Catalonia, Spain; University of Calgary, CANADA

## Abstract

The characterization of interactions between melatonin, one main ingredient of medicines regulating sleeping rhythms, and basic components of cellular plasma membranes (phospholipids, cholesterol, metal ions and water) is very important to elucidate the main mechanisms for the introduction of melatonin into cells and also to identify its local structure and microscopic dynamics. Molecular dynamics simulations of melatonin inside mixtures of dimyristoylphosphatidylcholine and cholesterol in NaCl solution at physiological concentration have been performed at 303.15 K to systematically explore melatonin-cholesterol, melatonin-lipid and melatonin-water interactions. Properties such as the area per lipid and thickness of the membrane as well as selected radial distribution functions, binding free energies, angular distributions, atomic spectral densities and translational diffusion of melatonin are reported. The presence of cholesterol significantly affects the behavior of melatonin, which is mainly buried into the interfaces of membranes. Introducing cholesterol into the system helps melatonin change from folded to extended configurations more easily. Our results suggest that there exists a competition between the binding of melatonin to phospholipids and to cholesterol by means of hydrogen-bonds. Spectral densities of melatonin reported in this work, in overall good agreement with experimental data, revealed the participation of each atom of melatonin to its complete spectrum. Melatonin self-diffusion coefficients are of the order of 10^−7^ cm^2^/s and they significantly increase when cholesterol is addeed to the membrane.

## Introduction

Cell membranes are biological structures composed of hundreds of different classes of lipids, sterols and proteins, acting as boundaries of cells [1]. The composition of a membrane can affect its fluidity and structure, so that addition of different molecules to the membrane may be able to change substantially its properties [2, 3]. Furthermore, the human cell membrane acts as an external selective container of the cell elements, so it is very important to know its structural and dynamical properties concerning new, external molecules appearing at the interface of membrane bilayer systems. For instance, recent studies have shown that the role of some proteins and their interactions with components of plasma membranes is extremely important to understand the mechanisms of protein anchoring at the membrane that can lead to oncogenesis [4].

In this work we have focused our efforts on the study of the binding of a small molecule, the neurohormone melatonin (MEL) [5, 6] at a simplified model cell membrane. This is a process that aims to improve our understanding of the basic mechanisms of molecular binding and crossing of biological membranes by small solutes and the interactions with their surroundings. Nevertheless, reproducing cell membranes of mammalians using realistic computational methods is a highly difficult task [7]. In particular, all-atom simulations involve the computation of interactions between *N* particles, where *N* is of the order of 10^5^, so that for a single run computational times scale as *N*(*N* − 1) and make the simulation a challenging task, often requiring the use of high-performance computational facilities. Given the cost of such realistic calculations involving a wide variety of components, well beyond the scope of the present work, we must assume some simplifications. One of most usual is to consider a single class of lipids. In the present work, we have considered a model membrane made with cholesterol and only one type of phospholipid, dimyristoylphosphatidylcholine (DMPC), extensively studied in the literature from the experimental and also computational points of view [8–10] and that belongs to the class of phosphatidylcholines, basic components of lecithin, a substance forming egg yolk and soy. Plenty of experimental and computational work on mixtures of cholesterol and melatonin at phosphatidylcholine membranes has been published to analyze the joint effects of the two species (see for instance [11, 12]), allowing us to ensure the reliability of our simulations since, as we will show below, the force field employed in the present work has revealed to be very successful in describing the physical properties of a DMPC membrane.

The benefits of MEL in the human body have recently drawn much attention in different fields. MEL is a natural hormone secreted by the pineal gland well known to regulate biological rhythms [13], to induce sleep [14], and that can also contribute to protect the organism from Alzheimer disease [15]. MEL is reported to induce/promote complex antioxidative and DNA repair systems which make it a very good candidate for curing several dermatoses associated with substantial oxidative damage. It helps for preventing skin cancer, skin photo- and radioprotection and also works as an inducer of repair mechanisms of human skin recovery from environmental damage [16, 17]. In recent years, the community of biologists, dermatologists and physicists have published plenty of works on MEL and studied how it can affect the human body [18–21]. For instance, MEL was found to have a significant effect on reducing cholesterol absorption and causing greatly decreases in total cholesterol in membrane bilayers and concentrations of cholesterol in the liver [22]. MEL is not only important for humans, but also for plants and animals. In particular, MEL works as a multifunctional signaling molecule which regulates broad aspects of responses to environmental changes [23].

In summary, the present study is devoted on the analysis of the structure and dynamics of melatonin and it follows previous works where tryptophan (the precursor of MEL) [24, 25] and other similar solutes were simulated [26]. In order to investigate these relevant effects of MEL on human body and more specifically on plasma membranes, we have focused our attention to the characterization of the structure and transport processes of MEL at the atomic scale with the aid of molecular dynamics (MD) simulations at the time-scale of hundreds of nanoseconds.

## Methods

There exist plenty of experiments and simulations already performed on DMPC and on similar phospholipids such as dipalmytoilphosphatidylcholine (DPPC), usually including the study of the influence of cholesterol [11, 12, 27–29]. These results will allow us to validate our methods and results, as it will be described in the beginning of Section “Results and Discussion”. Further, the transition temperature from gel to liquid crystal phase of DMPC has been determined and it is close to 297 K [30], what allows us to perform computer simulations able to be equilibrated in a reasonable short time. When transition temperatures are higher (case of DPPC, T = 314 K), we have previously observed that getting an equilibrated bilayer membrane will require much longer simulation runs. As a general fact, DMPC membranes have been satisfactorily reproduced in a wide variety of simulations and, in particular, the force field employed in the present work has revealed to be very successful in the reproduction of the structural characteristics of the DMPC membrane, as we will show below.

**Fig 1:**
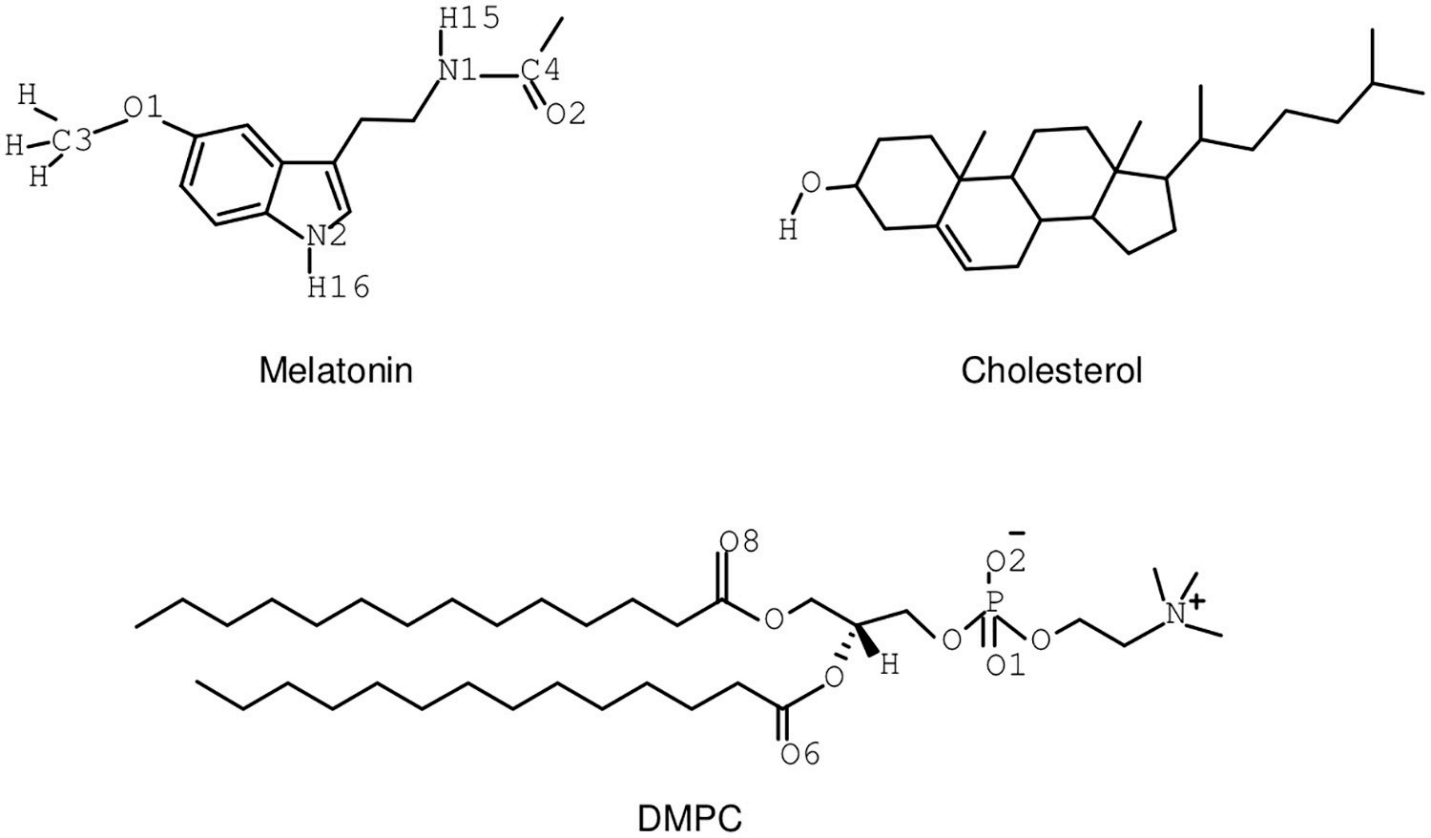
Backbone structures. Sketches of molecular structures of MEL, DMPC and cholesterol. Part of Hydrogen-Carbon bonds not shown. The highlighted sites of MEL (C3, C4, H15, H16, N1, N2, O1 and O2) and of DMPC (O1, O2, O6 and O8) will be referred in the text by the same labels.

A realistic model of DMPC-cholesterol membrane bilayers in a sodium chloride solution has been generated with the well-known CHARMM-GUI web-based tool [31, 32]. Cholesterol-free system was composed of 204 DMPC (*C*_36_*H*_72_*NO*_8_*P*) lipids molecules, approximately 10250 TIP3P [33] water molecules (allowing flexible bonds through harmonic springs), with 21 sodium and 21 chlorine ions of physiological concentration, along with one single MEL molecule (*C*_13_*H*_16_*N*_2_*O*_2_, obtained from pdb 4QOI through PDB Reader plugin of CHARMM-GUI website-based tool). From information previously obtained [25], only two significant cholesterol (*C*_27_*H*_46_*O*) percentages should be considered: 30, and 50% such that the harvested data should be compared with the results obtained for the case with 0% cholesterol. MEL locates at the interface of membrane bilayer in all three cases in the beginning of minimization. Sketches of the backbone structure of MEL, DMPC and cholesterol are represented in Fig 1. The NAMD2 software package [34] with the recently reparameterized CHARMM36m force field [35–38] was used in all MD simulations at a fixed temperature of 303.15 K and at the fixed pressure of 1 atm (NPT), in order to make sure all simulations were performed at the liquid crystal phase [39]. As usual in such kind of simulations [24] the temperature was regulated with a Langevin thermostat [40] with damping coefficient of 1 ps^−1^, whereas the pressure was controlled by a Nosé-Hoover Langevin piston [41] with Langevin dynamics [42] at an oscillation period of 50 fs.

All MD simulations were operated in NPT conditions. After 150 ps NPT relaxation and 100 ns equilibration periods, several production runs were generated and statistically meaningful trajectories of more than 100 ns were recorded in all cases. The simulation boxes had different sizes because of different cholesterol concentrations. For instance, the size of cholesterol-free system was of 79 Å × 79 Å × 85 Å. A time step of 2 fs was used and periodic boundary conditions were applied. All bonds involving hydrogens were set to fixed lengths, allowing fluctuations of bond distances and angles for the remaining atoms. During the calculation of spectral densities all bonds (including those involving hydrogens) were left flexible. The cutoff for the Van der Waals interactions was of 12 Å and a switching function was employed starting at 10 Å. Coulomb forces were computed using the particle mesh Ewald method [43], with a grid space of 1 Å. Every time step electrostatic interactions were updated. The usual periodic boundary conditions in all directions of space were taken.

## Results and discussion

### Physical characteristics of the membranes

**Fig 2:**
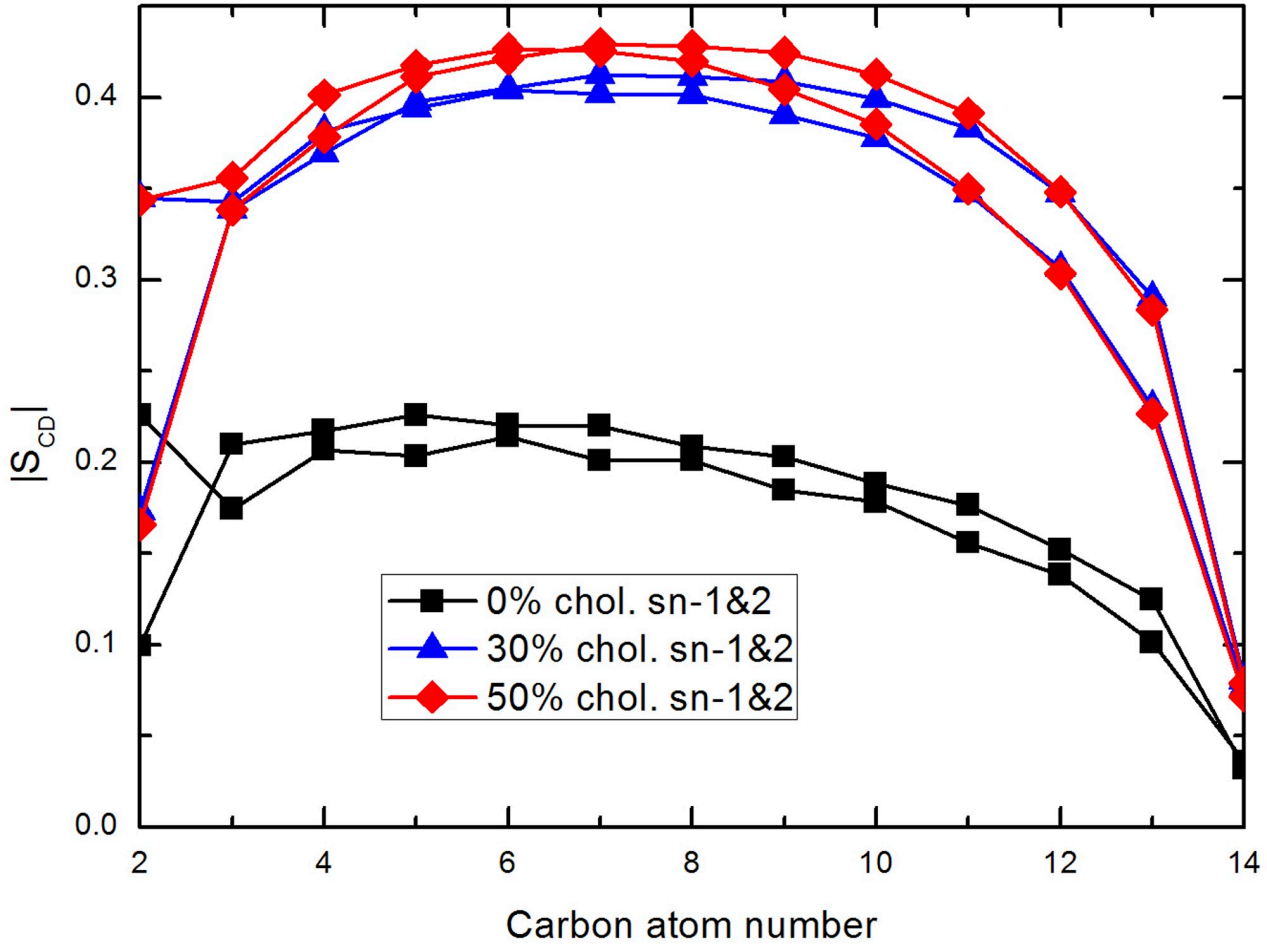
Order parameter. |*S*_*CD*_| for the (sn1, sn2) acyl tails of DMPC at three different cholesterol concentrations.

In order to explore the phase-diagram states of the model systems being simulated as well as to efficiently characterize the ordering inside the hydrated lipid bilayer, a procedure already employed in previous works [25, 28, 44, 45] was used. A deuterium order parameter *S*_*CD*_ was defined for each *CH*_2_ group of the DMPC lipid tails as follows:

$$\begin{array}{c} S_{CD}=\frac{1}{2}\left(3<\cos^{2}\theta_{CD}>-1\right), \end{array}$$ (1)

with *θ*_*CD*_ standing for the angle between the direction normal to the surface of the membrane and a CH-bond. *S*_*CD*_ can be also obtained from ^2^H NMR experiments [46]. The averaged results are shown in Fig 2 for both tail chains of all DMPC lipids at the three cholesterol concentrations considered in this work. The results for the cholesterol-free case were previously tested [28] and are in good agreement with both simulation [47, 48] and experimental works [49, 50], confirming the liquid crystal phase was represented well in the three systems adopted in the present work. We should note that as cholesterol concentration increases in the system, the tendency to higher ordering increases too, which is represented by profiles of *S*_*CD*_ having larger maxima (around ∼0.45 for the cholesterol-rich setups *versus* 0.25 for the cholesterol-free system), a tendency which was already observed by Petrache et al. [50].

**Fig 3:**
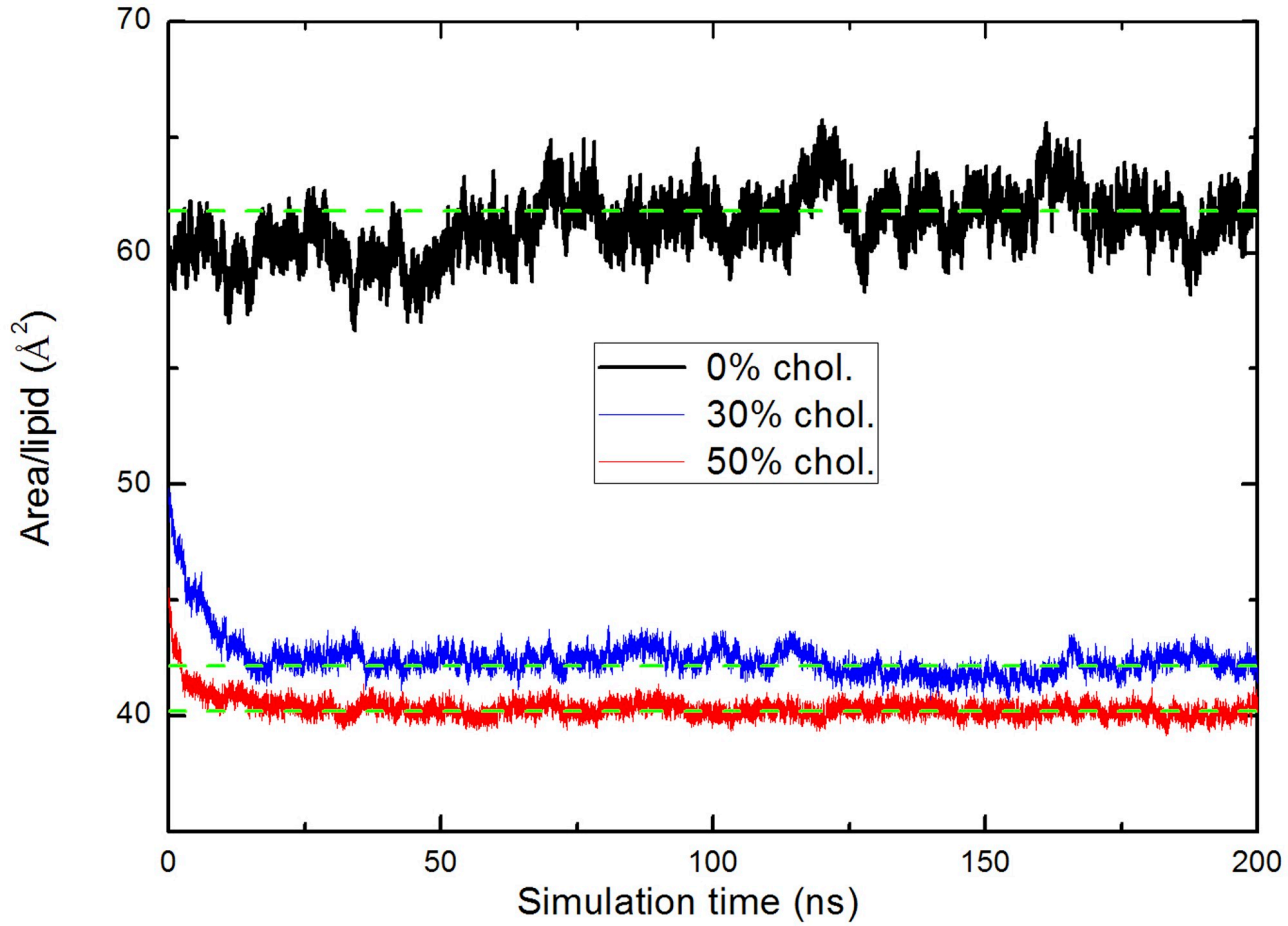
Physical characteristics of the membranes. Area per lipid of systems with different cholesterol contents: 0% (black line), 30% (blue line), 50% (red line) as a function of simulation time. The green dashed lines indicate the average values for the last 150 ns at each concentration.

The area per lipid is definitely a relevant output from most molecular simulation of plasma membranes. We have calculated the area per lipid considering the membrane surface along the *XY* plane divided by the number of lipids and cholesterol [51]. For continuous MD production runs, area per lipid as a function of simulation time is reported in Fig 3 whereas their averaged values together with the averaged thickness of membranes are reported in Table 1. Area per lipid decreases as cholesterol concentration increases. We obtained a value of around 62 Å^2^ for a cholesterol-free system and smaller values down to 40 Å^2^ for the system with a concentration of cholesterol of 50%. These results are in excellent agreement with other computational works [50, 52] where the value for pure DMPC is of about 60 Å^2^ at 303 K. According to the review of Nagle et al. [53], values of area per lipid of pure DMPC membranes (303 K) can be obtained from multiple methods (neutron scattering, X-ray and NMR) and were reported to be between 59 and 62 Å^2^ at the liquid phase. In our case, the change in the area per lipid has been observed to be more marked when the concentration of cholesterol was above 20% (not reported). This is consistent with the observed fact that DMPC membranes in this work experienced the phase transition point from a liquid-disordered phase (cholesterol-free system) to a liquid-ordered phase (systems of cholesterol 30% and 50%) [29, 54].

**Table 1:** Area per lipid and thickness of the membrane for all three cases studied in this work. *A* and Δ*z* at different cholesterol concentrations. Estimated errors in parenthesis.

Percentage of cholesterol	*A* (Å^2^)	Δ*z* (Å)
0%	61.8(1.2)	34.9(0.6)
30%	42.1(0.5)	44.3(0.3)
50%	40.2(0.3)	44.7(0.3)

Thickness of the membrane may provide additional clues about the influence of cholesterol on the mechanical properties of plasma membranes, such as rigidity and capability of allowing the movement of species in and out of the cell. We have obtained the thickness of the membrane Δ*z* by computing the distance between phosphorus atoms (P) of the DMPC head groups from both layers.

The results of the thickness of the membrane are in good agreement with those reported by Kučerka et al. [52] by means of X-ray and neutron scattering. These authors reported a value of 36.7 Å at 303 K for the DMPC membrane at a cholesterol-free system. In the present work we observe a tendency to larger bilayer thickness as cholesterol concentration increases. As it was pointed out in the case of the binding of tryptophan at DPPC-cholesterol membranes [25], at higher cholesterol percentages, the values of *A* are smaller: the larger the cholesterol contents the more compressed are the bilayer structures. This eventually can increase the rigidity of the membrane, extending the lipid tails and producing larger bilayer thickness. In summary, the increase of the rigidity of the membrane is a fact already observed by several authors from both experimental and computational sides, such as Drolle et al. [11] or Choi et al. [12] for cholesterol-melatonin mixtures in phosphatidylcholine membranes. In their studies these authors found out the effect of melatonin reducing the thickness of the membrane and enhancing its fluidity, a compensating effect of the condensation introduced by cholesterol. In the present work we only considered a single melatonin molecule what did not allow us to explore the joint effects of melatonin and cholesterol on the thickness of the membrane.

**Fig 4:**
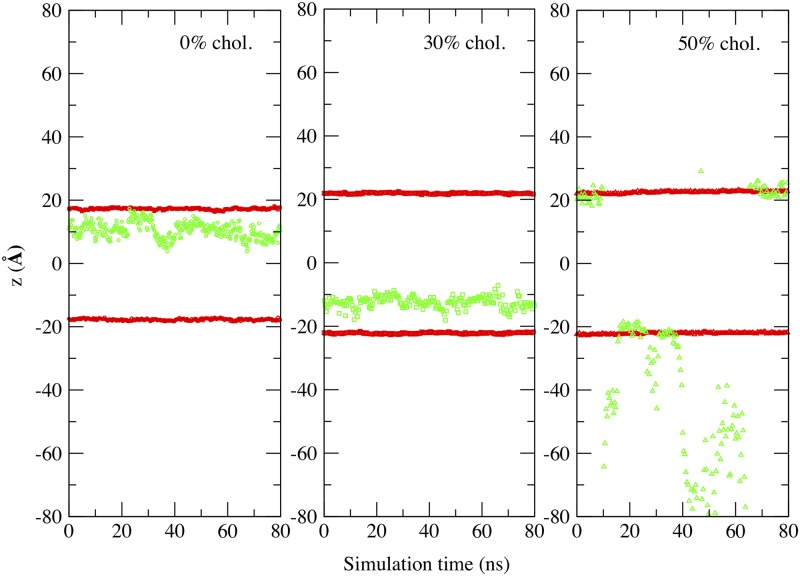
Z-axis position of MEL. Penetration of MEL inside DMPC bilayer (green symbols indicate the position of the center of mass of MEL whereas red symbols stand for the position of phosphorus atoms atoms in each layer) at 0%, 30% and 50% cholesterol concentrations.

The penetration of MEL in the membrane along its normal direction is also a relevant feature. We report in Fig 4 the Z-axis position of MEL from the center of the bilayer (i.e. *z* = 0) using the last meaningful 80 ns of each production trajectory in all three cases, namely, concentrations of cholesterol set at 0%, 30% and 50%. Red symbols represent averaged positions of phosphorus (P) atoms of the head groups of DMPC along the direction normal to the membrane (*Z*-axis); green symbols stand for the Z-axis distance between the center of mass of MEL and the center of DMPC bilayers, as a function of simulation time. It stands clearly that the thickness of the DMPC bilayer membrane is increased when cholesterol is present in the membrane, in good agreement with the results of thickness reported in Table 1. At 0% and 30% cholesterol systems, MEL stays in the internal region of DMPC bilayers most of the time, with a few occasional visits to the interface of the membrane, i.e. surroundings of the P atoms; however, when more cholesterol is added into the system (i.e. at 50%), MEL can diffuse into water bulk and become fully solvated by water or the head groups of DMPC without changing the size of system too much, indicating the competition between water and lipids to solvate MEL. These results are in good agreement with those of Drolle et al. that reported the preferential location of MEL in a DPPC bilayer at distances around *z* = 1.2 nm (see Fig 8 of Ref. [11]), i.e. at the crossover region between lipid head groups and the fatty acid chains. In the case of 50%, Fig 4 shows that MEL has the ability of either being adsorbed by the the head groups of DMPC either to stay for long periods of time in the water bulk, so we can expect that it is not difficult for a small molecule like MEL to cross the free energy barrier between the two states (solvated by water or solvated by the head groups of DMPC). This latter aspect will be addressed with more details below.

### Radial distribution functions of melatonin around DMPC, water and cholesterol

**Fig 5:**
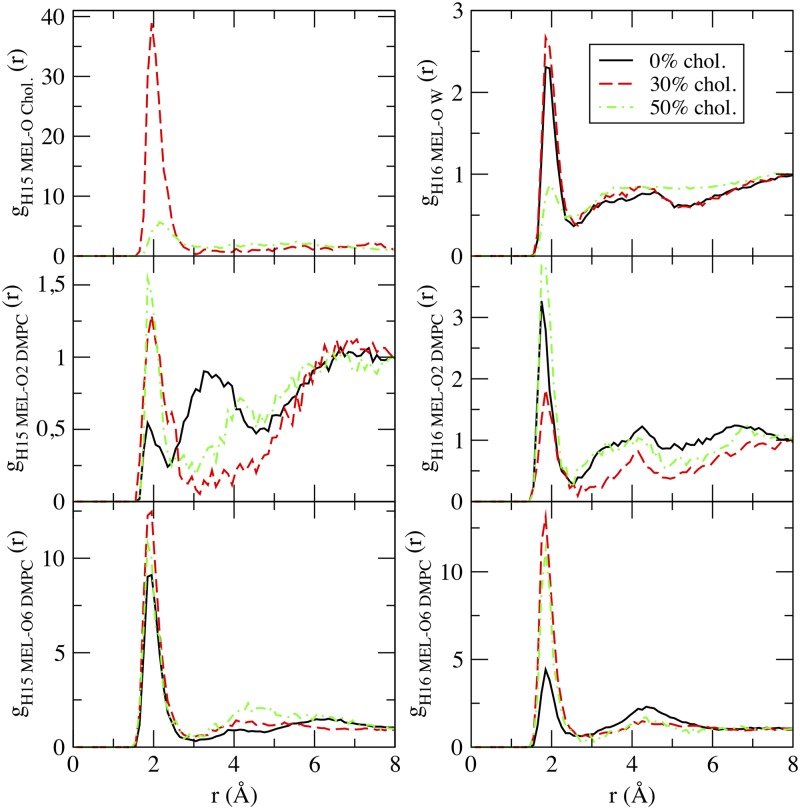
Radial distribution functions. Selected radial distribution functions for hydrogens of MEL (‘H15’ and ‘H16’) with oxygens of water (‘OW’); DMPC (‘O2’ (representing ‘O1&O2’) and ‘O6’ (representing ‘O6&O8’)) and cholesterol, belonging to hydroxyl group (‘O Chol’).

A direct route to the characterization of the local structure of each atomic species of the system is usually obtained by means of normalized radial distribution functions (RDF) *g*_*AB*_(*r*) for two different species *A* and *B* (see Eq (2) of Ref [25]). Among the wide variety of possible RDF that could be computed, we have considered only six relevant RDF based on the first coordination shells of ‘H15’ and of ‘H16’ of MEL. The remaining RDF indicate low maxima at distances significantly longer than the typical hydrogen-bonding values or show too noisy profiles which indicate that the corresponding local structures are not stable enough. The selected g(r)s are reported in Fig 5 for three cholesterol percentages (0, 30, 50%).

All six g(r)s show some fluctuations in their profiles, especially at the coordinates of *r* = 3 Å and beyond, i.e. those corresponding to second coordination shells. We could observe a neat first coordination shell in every case, located around 1.8-2.0 Å that should be essentially attributed to hydrogen-bonds (HB) between MEL and the remaining species, cause such distance is the signature of typical oxygen-hydrogen HB in water [55]. Interestingly, the largest peak in all RDF is, by far, the one appearing at the MEL-cholesterol association, centered at 1.9 Å when the concentration of cholesterol is of 30%. Further, when we raised the concentration to 50% such band decreased dramatically and its position was shifted to about 2.2 Å. The interaction of MEL with cholesterol in DPPC bilayers, already reported by Choi et al. [12] produced a fluidizing effect on the membrane for a melatonin concentration high enough, opposite to the condensing effect of cholesterol.

In all the remaining cases, HB lengths are around 1.9 Å. The height of each maxima (related to the intensity of the HB) depended strongly of the concentration of cholesterol, as follows: (1) in the case of MEL-water association, strong HB were observed between ‘H16’ and water at 0% and 30% although at 50% they were much weaker; (2) both ‘H15’ and ‘H16’ were able to form HB with the DMPC sites ‘O1’ (or ‘O2’, both sites sharing the negative charge); (3) finally, MEL can establish HB between both ‘H15’ and ‘H16’ hydrogens with the DMPC sites ‘O6’ (or ‘O8’) in all three percentages of cholesterol. These findings of HB association between MEL and DMPC are in good agreement with those from Severcan et al. [56] who, by means of Fourier transform infrared spectroscopy, observed the existence of hydrogen bonding between the hydrogen in the N-H group of the furanose ring of MEL (labeled ‘H16’ in the present work) and the carbonyl (C = O) and phosphate (${\text{PO}}_{2}^{-}$) groups in DPPC membranes. From our findings we have observed both HB of ‘H15’ and ‘H16’ of MEL with the phosphate group of DMPC (‘O1’) and also with the more internal C = O groups (‘O6’ and ‘O8’). Thus, the novelty here is the hydrogen bond association of ‘H15’ with the two well-known acceptor groups in phosphatidyl-cholines indicated above, together with the already reported association of ‘H16’. This fact allows MEL to be adsorbed deeper than tryptophan [25] at the membrane lipid bilayer with two selected donors (‘H15’ and ‘H16’) as well as through ‘H15-O Chol.’ bridges, which provides a variety of structures as it will be described in full details below. Results on MEL located close to the lipid head groups in studies of MEL inside DOPC and DPPC membranes were found by Drolle et al. [11] by means of small-angle neutron diffraction and MD simulations as well.

### Estimation of Helmholtz free energy differences

From a general perspective, the calculation of the Helmholtz or Gibbs free energy differences for binding processes or for configurational changes is a difficult task and it requires a considerable amount of computer time and a precise knowledge of the hypersurface of potential energy of the system [57]. This can be explored by means of methods such as metadynamics [58, 59], hybrid quantum mechanics/molecular mechanics methods [60] or transition path sampling [61–64]. However, a usual way to obtain free energy estimations is through the so-called potential of mean force (PMF), which is an estimation of the Helmholtz free energy difference between two particles (1, 2). Computing the reversible work required to move the two tagged particles from infinite separation to a relative separation *r*, PMF can be directly obtained from the pair (atom-atom) radial distribution function *g*_12_(*r*) as follows [26, 65]:

$$\begin{array}{c} W_{12}\left(r\right)=-\frac{1}{\beta }\ln g_{12}\left(r\right), \end{array}$$ (2)

where *β* = (*k*_*B*_*T*)^−1^ is the Boltzmann factor. The only restriction of this method is the fact that a single radial distance is always assumed as the pre-conceived reaction coordinate. Using this procedure on the RDF reported above, we have estimated the height of the barriers appearing in the Helmholtz free energy differences for MEL when bound to selected atomic sites from water, DMPC and cholesterol, assuming that at 303.15 K, the equivalence is *k*_*B*_*T* = 0.602 kcal/mol. The results are reported in Table 2:

**Table 2:** Helmholtz free energy differences *W*_12_(*r*) (in kcal/mol) for the binding of MEL (sites ‘H15’ and ‘H16’) to selected atomic sites. Since ‘O1’ and ‘O2’ sites in DMPC share the negative charge, their contributions have been averaged; the same situation for ‘O6’ and ‘O8’.

Atomic site	H15			H16		
	0%	30%	50%	0%	30%	50%
O-water	-	-	-	1.08	1.20	0.42
O2-DMPC	0.48	1.69	1.14	1.45	1.56	1.62
O6-DMPC	1.99	1.87	1.93	1.14	1.87	2.23
O-chol.	-	2.53	0.90	-	-	-

From the results in Table 2 we can observe that all Helmholtz free energy differences are of the order of 1 kcal/mol, in overall good agreement with preliminary calculations (case of a cholesterol-free DPPC membrane at 323 K) [26]. Density functional theory calculations of binding energy barriers of aqueous solvation of MEL in water clusters have been found to be of a few kcal/mol [66]. In most cases, a single coordination shell has been observed. As expected from the RDF reported above (see Fig 5), the highest free-energy barrier corresponds to the association of MEL with cholesterol, at the 30% concentration, where the hydrogen bonding distance is very close to 2 Å and tends to increase when cholesterol concentration is of 50%. Conversely, all remaining HB distances have been found at the typical value of 1.85 Å. MEL-water HB have been observed, in a significant amount, for ‘H16’ and not for ‘H15’ and they become very weak at the highest cholesterol concentration.

Focusing on DMPC-MEL HB, we have encountered strong influence of cholesterol, with overall highest barriers at 50% concentration. The typical bindings sites were ‘O1-O2’ (located at phosphate belonging to the head groups of DMPC) or ‘O6-O8’ (at carbonyl species on the tail groups of DMPC). Our results indicate that the presence of cholesterol increases the energetic cost of the binding of MEL to the membrane, with preferential association of MEL ‘H15’ to the phosphate group of DMPC and of ‘H16’ to carbonyl groups, all depicted in Fig 1. This is in agreement with the preferential location of MEL deep inside the 30% cholesterol membrane and also with the fact that MEL can move outside the membrane more easily as cholesterol concentration increases (see Fig 4 as well as data from Refs. [11] and [12]).

### Angular orientations of melatonin at the membrane

**Fig 6:**
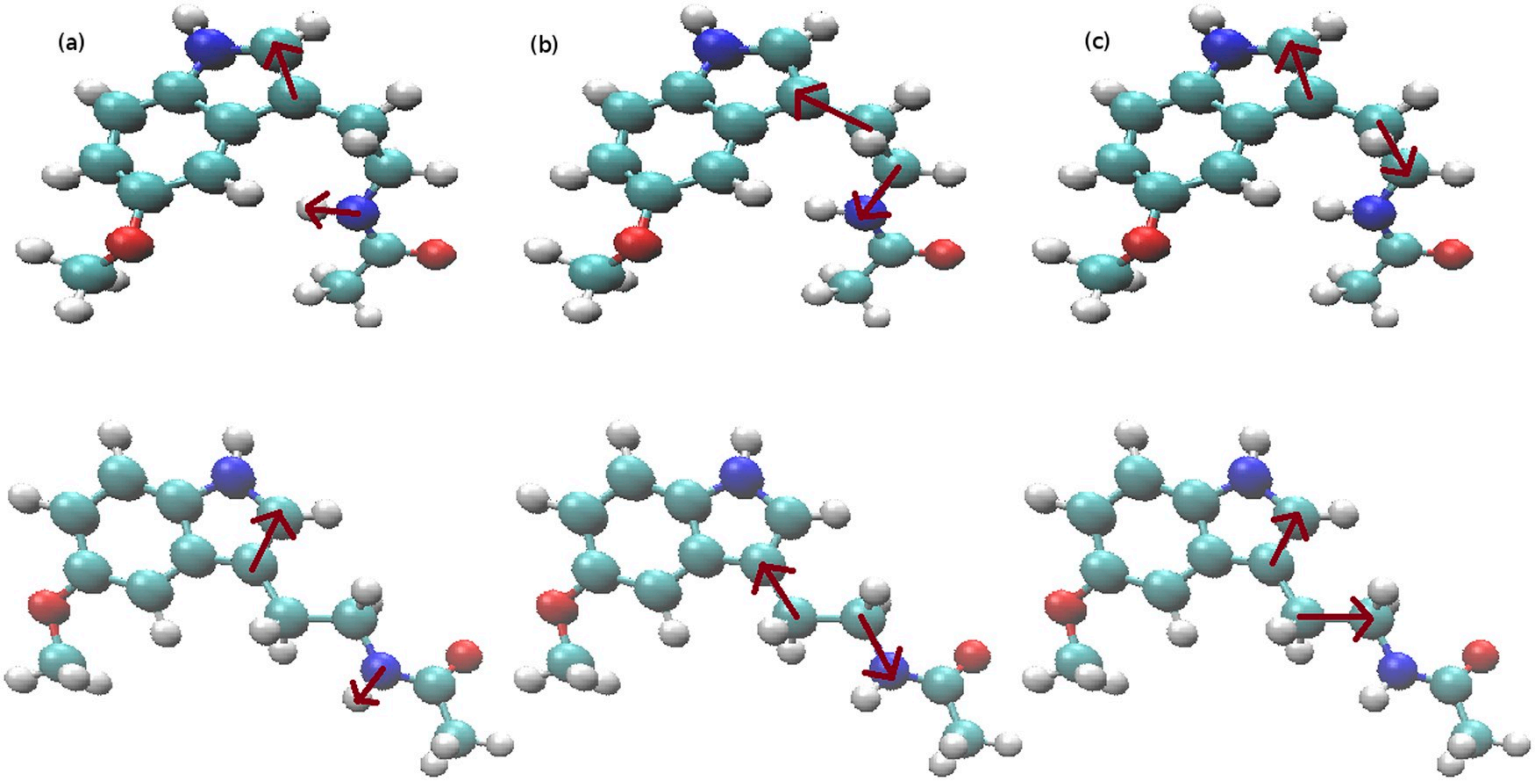
Dihedral angles. **Angles (a) *θ*, (b) *ψ* and (c) *ϕ* defined for MEL**. Top figures correspond to “folded” and bottom figures to “extended” configurations for each dihedral. The two snapshots (top, bottom) have been obtained from equilibrated configurations.

In order to analyze the angular distributions of MEL at the interface of the membrane, we have defined three sorts of angles, according to Fig 6.

**Fig 7:**
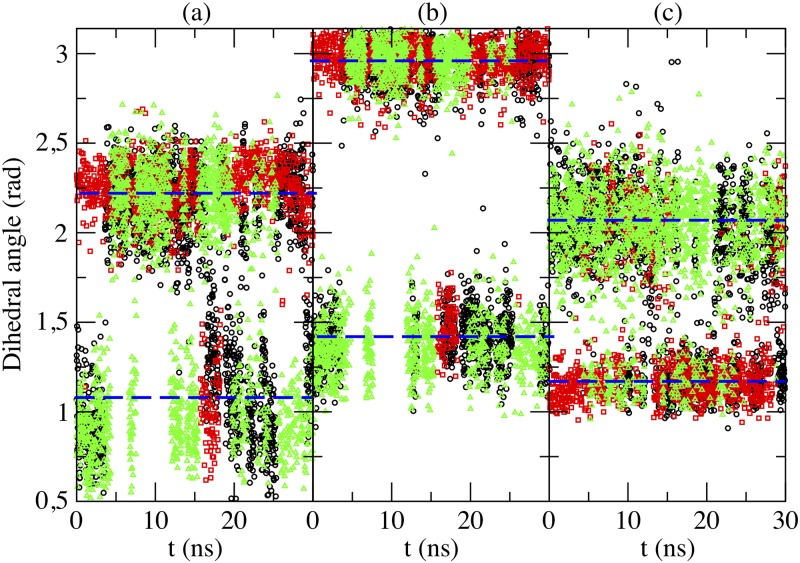
Angular distributions. Distribution of selected dihedral angles in MEL as a function of simulation time, where labels (a), (b) and (c) have the same meaning as in Fig 6. Percentages of cholesterol are: 0% (black circles), 30% (red squares), and 50% (green triangles). Dashed lines indicate average values and are a guide for the eye.

We report in Fig 7 angular distributions of MEL using the three selected dihedral angles defined in Fig 6. In all cases we can observe that, in average, the angular distributions of MEL are centered around two preferential orientations, called “folded” and “extended” configurations, that are found at all cholesterol concentrations. The nitrogen atom involved in dihedrals *θ* and *ψ* is the one labeled ‘N1’ in Fig 1, namely the nitrogen chemically bound to the hydrogen labeled ‘H15’ in Fig 1. For the dihedral angle *θ*, the average angles corresponding to stable configurations are of 1.08 rad (62°, folded configuration) and 2.22 rad (127°, extended configuration); for angle *ψ*, the two stable configurations correspond to 1.42 rad (81°, folded) and 2.96 rad (170°, extended), whereas for *ϕ* the values are of 2.07 rad (119°, folded) and 1.17 rad (67°, extended).

From the distributions reported in Fig 7, we can note that the dihedral angle *θ* can reach continuously all sorts of values between 0.44 and 2.70 rad, the angle *ϕ* can fluctuate between 0.98 and 2.60 rad, whereas the angle *ψ* is much better defined and it reaches either values around 2.96 ± 0.18 rad or 1.42 ± 0.41 rad, regardless of the concentration of cholesterol of the system. After analyzing 100 ns of equilibrated trajectories (production runs), we calculated the ratios of the three dihedral angles for equilibrated systems with different cholesterol concentration and reported the averages in Table 3.

**Table 3:** Ratio of the two angular configurations for MEL in three systems with different cholesterol concentration. “Ext.” stands for “extended configuration of MEL” and “Fol.” stands for “folded configuration of MEL” in all cases.

	*θ*		*ψ*		*ϕ*	
	Ext.	Fol.	Ext.	Fol.	Ext.	Fol.
0%	0.53	0.47	0.46	0.54	0.40	0.60
30%	0.53	0.47	0.52	0.48	0.46	0.54
50%	0.62	0.38	0.56	0.44	0.19	0.81

From the results of dihedral angles *θ* and *ψ* (see Table 3), we get that the extended configuration of MEL is more favored when adding cholesterol into the system. However from the results of *ϕ*, it seems that at 50% cholesterol percentage, MEL stays much less at its extended configuration than 0% and 30%. Considering the behavior of MEL in all three cases (see Fig 4), we found that MEL spent almost half of the simulation time in the water bulk at the 50% cholesterol system and it stayed at the interface for 0% and 30% cholesterol systems. So, we can suggest that the dihedral angle *ϕ* is significantly more sensible than the other two dihedral angles as being solvated by water. We can also suggest that introducing cholesterol into the system could help MEL change from its folded to its extended configuration more easily through hydrogen-bonding between MEL-DMPC and MEL-cholesterol. Also, according to this, *ψ* is an excellent candidate for being used as a collective variable in metadynamics calculations [58, 67] of free energy landscapes for MEL binding in biomembranes. We are currently performing such type of calculations in our laboratory. Preliminary results confirm the suitability of *ψ* as a collective variable.

### Dynamics properties: Diffusion and vibrational spectra

#### Diffusion of melatonin and water

Dynamics of MEL and water has been extensively explored through translational diffusion and vibrational spectroscopy, as we will report below. Conversely, dynamics of lipids and cholesterol is much slower and it has not been considered here. In particular, the mean square displacements (MSD) of water and center of mass of MEL have been evaluated. From the long time slopes of MSD (not reported here), we computed the corresponding self-diffusion coefficients using Einstein formula (see Eq (3) in Ref [25]). The results are reported and summarized in Table 4.

**Table 4:** Self-diffusion coefficients *D* (in cm^2^/s) of MEL and of water in three systems with different cholesterol percentages. Estimated errors in parenthesis.

*D*	0%	30%	50%
Water	4.0(0.1)×10^−5^	4.3(0.1)×10^−5^	4.4(0.1)×10^−5^
MEL	1.1(0.4)×10^−7^	3.9(0.6)×10^−7^	4.1(0.9)×10^−7^

For the self-diffusion of water, all water molecules in the system are included in the results regardless of their location at bulk or interfacial regions. At 0% cholesterol our results are in overall agreement with the ones obtained by our team for the simple aqueous DMPC membrane [28] (2.7×10^−5^) what indicates that when cholesterol concentration rises, water tends to diffuse slightly faster. Nevertheless we can observe that the presence of cholesterol and the single MEL molecule does not affect the dynamics of water to a large extent. This could be due to the fact that with higher cholesterol concentration in the system plasma membranes are more packed and water cannot easily penetrate the interface of the membrane, having its main diffusion along the instantaneous surface of the bilayer.

Diffusion coefficients for MEL are approximately two orders of magnitude smaller than those of water and show a tendency to increase when cholesterol is mixed with DMPC, regardless of its concentration. In Table 4, at 30% cholesterol the value of *D* for MEL is six times larger than the value of *D* of DMPC molecules in pure DMPC bilayer membrane systems [28] (0.6×10^−7^ cm^2^/s), although the former are about a factor 6.5 larger. Thus, the diffusion of MEL is significantly faster than that of DMPC. This fact would suggest that its mechanisms of diffusion may be similar to those of an individual particle (such as in Fickian diffusion) and qualitatively different of those of lipids, whose diffusion was observed to occur in a sort of collective way, associated in local groups of a few units (around 5-10 units) [28].

#### Spectral densities of melatonin

**Fig 8:**
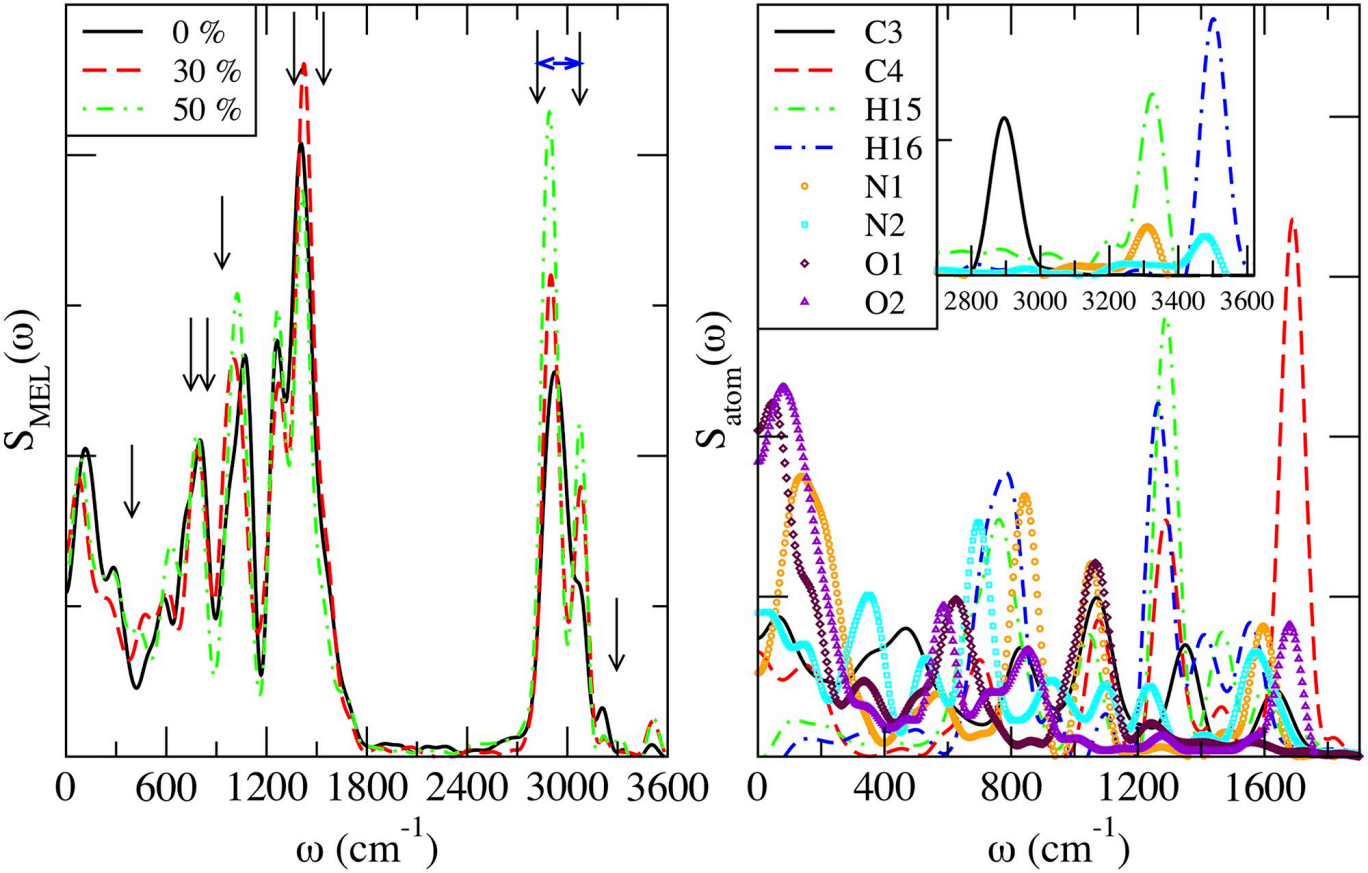
Spectral densities. *S*_*MEL*_(*ω*) of MEL (left). Positions of main experimental peaks reported in Ref. [71] are indicated by full arrows (low-mid frequency range) and locations of a broad group of frequencies from Ref. [72] (high frequency range) have been indicated with initial and final vertical arrows linked by a horizontal double arrow. For the cholesterol-free system (right), atomic spectra *S*_*i*_(*ω*) indicate the relative contribution of selected atoms to *S*_*MEL*_(*ω*). Inset: high frequency region for most relevant atoms of MEL (‘C3’, ‘N1’, ‘N2’, ‘H15’ and ‘H16’).

Infrared (IR) spectroscopy exploits the fact that molecules absorb specific frequencies that are characteristic of their structure and atom-atom interactions. IR spectrum is usually obtained on a spectrometer using the attenuated total reflection sampling technique with a neat sample in the laboratory. Such experimental properties could also be obtained from certain MD simulations [68, 69], and be directly related to the absorption lineshape *I*(*ω*). In many cases this physically relevant property known as the atomic spectral density *S*_*i*_(*ω*) is defined as [70]:

$$\begin{array}{c} S_{i}\left(\omega \right)=\int_{0}^{\infty }dt\;C_{i}\left(t\right)\cos\left(\omega t\right)\; \end{array}$$ (3)

where $C_{i}\left(t\right)=<{\vec{v}}_{i}\left(t\right){\vec{v}}_{i}\left(0\right)>$ is the velocity autocorrelation function for atom *i* and the brackets 〈⋯〉 denote equilibrium ensemble average. In this work we have obtained the spectral density of MEL and also of each atom site (only part of them are shown). Since the force field employed in the present work accounts for harmonic bond vibrations, we were able to locate most of the positions of main experimental spectral bands (see Ref. [25] for more details). At this point we should remark that one powerful characteristic of computing molecular spectra in simulations is the possibility of locating the particular atomic sites contributing to each spectral band and thus be able to make a precise interpretation of experimental spectra. We will report this feature below. The full spectral density *S*_*MEL*_(*ω*) and its decomposition into atomic contributions for the 0% cholesterol case is reported in Fig 8.

From left side of Fig 8, two main spectral regions of the full IR spectrum of MEL have been located: (1) frequencies below 1800 cm^−1^ and (2) frequencies of 2700 < *ω* < 3600 cm^−1^. As a general feature, the agreement of the calculated full spectrum with available data (at 0% cholesterol concentration) from infrared and Raman spectroscopy reported by Singh et al. [72], Fleming et al. [73] and Pieta et al. [71] have revealed a very good overall agreement (see left side of Fig 8), although some discrepancies have been observed in the location of some peaks. As a general fact, the maxima reported by the three experimental groups match each other very well. Let us describe the comparison of experimental data with results from the present work as follows.

In the region up to 1800 wavenumbers, the maxima with strong signatures were located at 401, 756, 834, 926, 1353 and 1550 cm^−1^, according to the recent measurements of Pieta et al. [71], whereas Fleming et al. [73] reported strong maxima at 404, 508, 836, 928, 1358, 1449 and 1553 cm^−1^. Most of these maxima are included in the *S*(*ω*) showed in the present work (Fig 8) at 0% cholesterol content. We observed maxima at: 120, 294, 592, 714 (shoulder), 813, 1077, 1265 and 1408 cm^−1^. The main disagreements are related to frequency shifts in most bands:

1. The peaks observed by us at 120 and 592 cm^−1^ are not seen in experimental data (the Raman frequency range started at around 250-300 wavenumbers in all cases);
2. The Raman band located at ∼ 400 cm^−1^ is found at ∼ 300 cm^−1^ in our spectrum;
3. The Raman bands at 756 and 834 wavenumbers are red-shifted down to 714 and 813 cm^−1^ in Fig 8;
4. The Raman band centered at 926 wavenumbers has been found around 1077 cm^−1^ in Fig 8;
5. The Raman bands at 1353 and 1550 cm^−1^ are also red-shifted to 1265 and 1408 cm^−1^ in the computed spectra.

In the high frequency region (2700 < *ω* < 3600 cm^−1^) the strong maxima reported from infrared spectroscopy measurements [72] were located at 3280 and 3302 cm^−1^ (assigned by the authors to the N-H stretching region), whereas a group of thirteen bands were located at the C-H stretching region, between 2826 and 3079 wavenumbers. The computed spectra of Fig 8 reveal four bands, one of them very strong at 2930 cm^−1^, another as a shoulder around 3070 cm^−1^ and two weak bands at 3210 and 3500 cm^−1^.

According to the overall agreement between our results and those from Raman and infrared data, we can assume a reasonably good reliability of the potential MEL model and method adopted in this work. Accordingly, we have computed the partial spectra of each atom in MEL, in order to identify and assign the microscopical participants and type of each vibrational mode. The results are depicted in the plot at the right side of Fig 8. There we computed the contribution of each individual atom to the full spectrum at 0% cholesterol concentration. The list of assignments and their physical meaning is as follows:

1. The peak located at 120 cm^−1^ in the computed spectra is directly related to oxygens ‘O1’ and O2’ as well as nitrogen ‘N1’, since it appears in the corresponding spectral densities. From previous knowledge [25, 28, 74] it should be attributed to restricted translations of the full MEL molecule.
2. The weak band located around 294 cm^−1^ along with the maxima at 345 cm^−1^ are approximately assigned to the spectrum of ‘N2’. Since this is a relatively low frequency it will probably correspond to a rotational motion of the indole group of the MEL molecule.
3. The maximum at 592 cm^−1^ directly matches a maximum of ‘O2’ and this suggests a librational motion of the ‘C4-O2’ bond.
4. The maxima at 714 cm^−1^ (shoulder) and 813 cm^−1^ (attributed to the experimental bands at 756 and 834 wavenumbers) are clearly connected to ‘N-H’ vibrational bending motions, since they are detected as maxima in the spectra of ‘N2-H16’ and ‘N1-H15’ pairs, respectively.
5. The band centered at 1077 cm^−1^ is matched in our detailed spectra by maxima of atomic pairs ‘N1-C4’ and ‘O1-C3’. Being this a bending-like mode, it should account for scissoring vibrations of the mentioned pairs.
6. The maxima at 1265 and 1408 cm^−1^ are observed in the partial spectra of ‘C3’ and ‘C4’, suggesting a stretching vibration of both carbons, the lowest frequency band associated with ‘C4’ and the highest to ‘C3’.
7. In the high-frequency range, the strongest band observed by us and located at 2930 cm^−1^ is well matched by the peak of ‘C3’ (see inset of *S*_*atom*_) and, according to previous works [25, 28] it indicates a stretching vibration of hydrogens bound to ‘C3’.
8. The shoulder at 3070 cm^−1^ can only be (hardly) seen at the spectral densities of ‘H15’ and ‘N1’.
9. Finally, the two weak maxima located around 3210 and 3500 cm^−1^ should be respectively attributed to the pairs ‘N1-H15’ and ‘N2-H16’ as stretching vibrational modes of the corresponding hydrogens.

When cholesterol is included in the system, we can observe at the left side of Fig 8 that some observable frequency shifts are produced, suggesting that cholesterol is able to interact with MEL in a remarkable way, affecting the vibrational motions of their atomic components. This is in good agreement with the fact reported above (see section 3.1) of the hydrogen-bonding of MEL to cholesterol, especially at the 30% concentration.

## Conclusion

We present all-atom molecular dynamics simulations of a bilayer membrane made up with the zwitterionic phospholipid DMPC at three cholesterol concentrations (0%, 30% and 50%) including a single MEL molecule, all in aqueous NaCl ionic solution at 303 K and at the fixed pressure of 1 atm. We adopted the most recent version of the CHARMM36m force field and the simulation length of each system reached the scale of hundreds of nanoseconds.

Our main interest was focused on the local structure and angular distributions of MEL, especially when associated to DMPC and cholesterol molecules. After this, Helmholtz free energy differences of MEL binding onto different membrane bilayer interfaces has been evaluated through potentials of mean force. A one-dimensional reaction coordinate based on radial atomic distances for selected atoms was considered. As a general fact, MEL is able to establish hydrogen-bonds with water, cholesterol and DMPC lipids. The strongest association has been observed between species ‘H15’ of MEL and the oxygen pertaining to the hydroxyl group of cholesterol; between ‘H15’ and the carbonyl groups in DMPC and between ‘H16’ and the carbonyl groups of DMPC, always when cholesterol was present in the system. However, some binding between ‘H15’ and ‘H16’ and the phosphate group in DMPC has been also observed. The order of magnitude of the Helmholtz free energy differences has been of 1 kcal/mol, with values between 0.5 and 2.5 kcal/mol. The typical hydrogen-bond distances have been found between 1.8 and 2.0 Å.

Two relevant MEL structures have been observed from angular distributions: “folded” and “extended” configurations. We defined three different dihedral angles to account for the two preferential angular configurations. One of them (dihedral *ψ* in Fig 6) has revealed to be a meaningful order parameter (i.e. may act as reliable reaction coordinate) to describe the dynamics of MEL, with preferential angles of ∼ 1.42 rad and ∼ 2.96 rad, as defined in Fig 6. From our results, we suggest that introducing cholesterol into the system could help MEL change from its folded configuration to extended configuration more easily, using hydrogen-bonds between MEL-DMPC and MEL-cholesterol.

The self-diffusion coefficient of MEL obtained from slopes of MSD at long times was found to be of the order of 10^−7^ cm^2^/s and the presence of cholesterol in the system has an influence on it. As cholesterol concentration increases the membrane tends to become progressively thicker and area per lipid is significantly reduced resulting that the diffusion of MEL increases a factor four. Spectral densities computed in this work are in overall good agreement with experimental Raman and infrared data [71–73] and have revealed the degree of participation of each atomic site of MEL to complete its whole molecular spectrum giving some clues to understand the microscopic origin of molecular vibrations and also giving evidence of the good reliability of the model we adopted in the present work.
